# Novel BRCA1–PLK1–CIP2A axis orchestrates homologous recombination-mediated DNA repair to maintain chromosome integrity during oocyte meiosis

**DOI:** 10.1093/nar/gkae1207

**Published:** 2024-12-09

**Authors:** Crystal Lee, Jeong Su Oh

**Affiliations:** Department of Integrative Biotechnology, Sungkyunkwan University, 2066 Seobu-ro, Suwon 16419, South Korea; Department of Integrative Biotechnology, Sungkyunkwan University, 2066 Seobu-ro, Suwon 16419, South Korea

## Abstract

Double-strand breaks (DSBs) are a formidable threat to genome integrity, potentially leading to cancer and various genetic diseases. The prolonged lifespan of mammalian oocytes increases their susceptibility to DNA damage over time. While somatic cells suppress DSB repair during mitosis, oocytes exhibit a remarkable capacity to repair DSBs during meiotic maturation. However, the precise mechanisms underlying DSB repair in oocytes remain poorly understood. Here, we describe the pivotal role of the BRCA1–PLK1–CIP2A axis in safeguarding genomic integrity during meiotic maturation in oocytes. We found that inhibition of homologous recombination (HR) severely impaired chromosome integrity by generating chromosome fragments during meiotic maturation. Notably, HR inhibition impaired the recruitment of CIP2A to damaged chromosomes, and the depletion of CIP2A led to chromosome fragmentation following DSB induction. Moreover, BRCA1 depletion impaired chromosomal recruitment of CIP2A, but not vice versa. Importantly, the impaired chromosomal recruitment of CIP2A could be rescued by PLK1 inhibition. Consequently, our findings not only underscore the importance of the chromosomal recruitment of CIP2A in preventing chromosome fragmentation, but also demonstrate the regulatory role of the BRCA1–PLK1–CIP2A axis in this process during oocyte meiotic maturation.

## Graphical Abstract

**Graphical Abstract:**
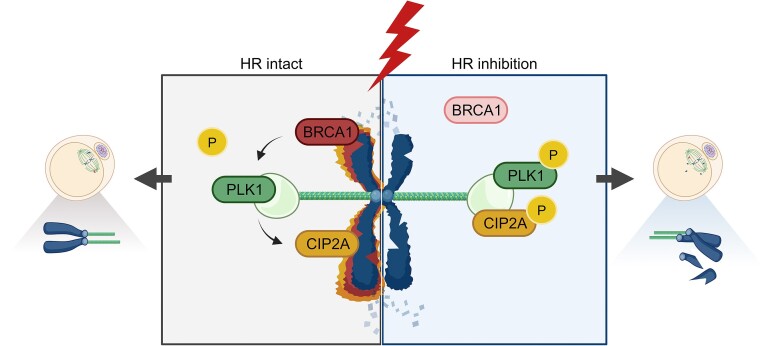

## Introduction

DNA damage poses a formidable threat to genome integrity, potentially leading to cancer and various genetic diseases (1). Among the diverse types of DNA damage, double-strand breaks (DSBs) are the most deleterious, as they can often profoundly alter genetic information if not faithfully repaired (2,3). To counteract such damage, cells have evolved intricate mechanisms collectively known as the DNA damage response (DDR). Central to the DDR is the activation of the ataxia-telangiectasia mutated (ATM) kinase in response to DSBs. ATM phosphorylates histone H2AX at Ser139, creating γH2AX (4). This modification acts as a molecular beacon, recruiting the scaffolding protein mediator of DNA damage checkpoint protein 1 (MDC1). Once phosphorylated by ATM, MDC1 further facilitates the recruitment of additional DDR factors, including ATM itself, leading to the spread of γH2AX signaling across neighboring chromatin. Moreover, MDC1 interacts with ring finger protein 8, promoting the ubiquitination of γH2AX and subsequent recruitment of downstream repair factors such as breast cancer gene 1 (BRCA1) and p53-binding protein 1 (5).

In mammalian cells, the non-homologous end joining (NHEJ) and homologous recombination (HR) pathways are primary for DSB repair (2). NHEJ directly rejoins broken ends, albeit with a tendency for errors, whereas HR uses a homologous strand as a template for error-free repair (6,7). DNA-dependent protein kinase (DNA-PK), DNA ligase IV and XRCC4 are critical components of the NHEJ, while RAD51-mediated strand exchange is crucial for HR (5–7). In addition, polymerase theta (POLQ)-dependent microhomology-mediated end joining (MMEJ), an error-prone repair mechanism that involves alignment of microhomologous sequences internal to the broken ends before joining, has recently emerged as a third player in DSB repair (8,9). However, the interplay among these pathways remains incompletely understood.

Mammalian oocytes arrest at the diplotene stage of the meiotic prophase I, referred to as the germinal vesicle (GV) stage, where they remain for an extended period. Following a hormonal surge, oocytes resume meiotic maturation, undergoing GV breakdown (GVBD) and progressing through metaphase I (MI) and metaphase II (MII), ultimately achieving meiotic maturity (10). Due to their extended lifespan, they are particularly vulnerable to the accumulation of DNA damage, necessitating efficient repair mechanisms to preserve genome integrity before fertilization (5,11). Unlike somatic cells, which are typically refractory to DNA repair during mitosis, oocytes are capable of undertaking DNA repair during meiosis (12–17). We previously demonstrated that oocytes selectively use NHEJ and HR to repair DSBs during meiotic maturation (15). Inhibition of NHEJ increases DNA damage levels and impairs the meiotic maturation of oocytes with DSBs by activating the spindle assembly checkpoint. Conversely, HR inhibition following DSB induction leads to chromosome fragmentation without affecting meiotic progression (15). However, the mechanism behind these fragmentations remains unknown.

Chromothripsis is a peculiar process that involves the fragmentation or pulverization of chromosomes and the subsequent reassembly of chromosomal regions (18,19). It occurs in response to severe cellular stress or catastrophic events, causing one or more chromosomes to shatter and subsequently reassemble in a seemingly random order (19). Recent studies have demonstrated that the cancerous inhibitor of protein phosphatase 2A (CIP2A) complexed with topoisomerase IIβ-binding protein 1 (TOPBP1) is responsible for clustering and tethering DNA fragments together, which prevents the loss of DNA pieces (20,21). Inactivation of CIP2A or TOPBP1 causes pulverized chromosomes to untether and disperse throughout the mitotic cells (20). Moreover, the intricate interplay of the CIP2A–MDC1–TOPBP1 complex has been found to not only tether fragmented chromosomes, but also activate pathways for DNA repair (22,23). Cells lacking CIP2A are unable to properly stabilize and/or repair DSBs during mitosis, leading to the accumulation of acentric chromosome fragments and consequent chromosome instability (23). Importantly, the significance of CIP2A in DNA repair extends beyond mitotic cells. Our previous study has demonstrated that the CIP2A–MDC1–TOPBP1 complex localizes at the spindle poles but is recruited to chromosomes after DNA damage, and depletion of CIP2A impairs DSB repair during meiotic maturation in oocytes (16). Notably, centromeres appear to play a critical role in this process, as depletion of centromeric protein A (CENP-A) impairs chromosomal recruitment of the CIP2A–MDC1–TOPBP1 complex and DSB repair. Moreover, BRCA1 localizes and enriches at the centromeres after DNA damage (16,24). However, the precise role of CIP2A and centromeres in DSB repair and their regulation in oocytes remain elusive.

In this study, we investigated whether chromosome fragmentation after inhibiting HR is associated with chromosomal recruitment of CIP2A during meiotic maturation in oocytes. We found that HR inhibition impaired CIP2A recruitment onto chromosomes in response to DNA damage. Moreover, CIP2A depletion led to chromosome fragmentation after DSB induction. Furthermore, BRCA1 depletion impaired chromosomal recruitment of CIP2A, which was rescued by inhibiting PLK1 activity. Thus, our study demonstrates the essential role of the BRCA1–PLK1–CIP2A axis in preventing chromosome fragmentation during meiotic maturation in oocytes.

## Materials and methods

### Oocyte collection and culture

Experiments with mice were approved by the Institutional Animal Care and Use Committee of Sungkyunkwan University (IDs: SKKUIACUC2023-10-39-1 and SKKUIACUC5 2024-02-25-1). Three-week-old female ICR mice (Koatech, South Korea) were primed with 5 IU pregnant mare serum gonadotropin 46–48 h before collection, and their ovaries were used for oocyte isolation at the GV stage. Oocytes were processed in M2 medium (Sigma–Aldrich, M7167) and supplemented with 100 μM 3-isobutyl-1-methylxanthine (IBMX; Sigma–Aldrich, I5879) to prevent meiotic resumption during preparation. To obtain oocytes at GVBD, MI and MII stages, GV oocytes were cultured *in vitro* in IBMX-free medium for 4, 8 and 15 h, respectively, under mineral oil (Irvine Scientific, 9305) at 37°C in a 5% CO_2_ environment.

### Chemical treatment

To induce DSBs, oocytes were treated with 50 μg/ml etoposide (ETP; Sigma–Aldrich, E1383) for 15 min. HR was inhibited by targeting RAD51 with 50 μM B02 (Tocris, 6392) (25). NHEJ was inhibited by targeting either DNA ligase IV with 20 μM SCR7 (Sigma–Aldrich, SML1546) or DNA-PK with 50 μM NU7441 (Selleckchem, S2638), respectively (26,27). MMEJ was inhibited by targeting POLQ with 5 μM ART558 (MedChemExpress, HY-141520) (28). For RAD52 inhibition, oocytes were treated with 50 μM D-I03 (MedChemExpress, HY-124691) (29). For PLK1 inhibition, oocytes were treated with 200 nM BI2536 (Selleckchem, S1109). All chemicals were dissolved in dimethyl sulfoxide (DMSO) and used at a dilution of 0.1% or less. Control oocytes were treated with an equal volume of DMSO.

### CIP2A and BRCA1 depletion

Acute depletion of CIP2A and BRCA1 was carried out using the Trim-away method as described previously (30,31). Briefly, GV oocytes were injected with Trim21-mCherry messenger RNA (mRNA) and incubated for 1 h in IBMX-supplemented M2 medium to allow protein expression. Oocytes were then matured in IBMX-free M2 medium for 5 h before an injection of CIP2A (Santa Cruz, sc-80662), BRCA1 (Abcam, ab17680) or IgG (Santa Cruz, sc-2027) antibodies at a final concentration of 100 ng/μl. After an incubation of 1 h, DSBs were induced with 50 μg/ml ETP for 15 min. Following ETP washout, oocytes were either fixed immediately at the MI stage or incubated until the MII stage for subsequent immunostaining or chromosome spreading.

### Immunostaining

Oocytes were fixed in 4% paraformaldehyde in phosphate-buffered saline (PBS) for 10 min, permeabilized in PBS containing 0.1% Triton X-100 and 0.01% Tween 20 for 30 min, and then blocked in 3% bovine serum albumin (BSA) in PBS for 1 h at room temperature. Oocytes were incubated overnight at 4°C with primary antibodies and then for 2 h at room temperature with secondary antibodies. Three rounds of washing, each for 10 min, were carried out after each antibody incubation. Chromosomes were counterstained with DAPI and examined using an LSM 900 laser scanning confocal microscope (Zeiss) with a C-Apochromat 63×/1.2 oil immersion objective.

### Chromosome spreading

After washing with fresh M2 medium, oocytes at MI or MII stages were exposed to acidic Tyrode’s solution (pH 2.5; Sigma–Aldrich, T1788) for up to 2 min to remove the zona pellucida and then fixed on a glass slide in 1% paraformaldehyde in PBS containing 0.15% Triton X-100 and 3 mM dithiothreitol. Oocytes on the slide were slowly dried in a humid chamber at 42°C for 1 h and then blocked with 3% BSA in PBS for 1 h at room temperature. Primary and secondary antibody incubations were carried out with a similar method to that used in immunostaining. Chromosomes were counterstained with DAPI and examined using an LSM 900 laser scanning confocal microscope (Zeiss) with a C-Apochromat 63×/1.2 oil immersion objective.

### TUNEL assay

To detect DSBs, terminal deoxynucleotidyl transferase dUTP nick end labeling (TUNEL) assay was conducted as described previously (16). Briefly, after preparing chromosome spreads as described earlier, the oocytes were incubated with fluorescent-conjugated terminal deoxynucleotide transferase dUTP for 2 h at 37°C using an *In Situ* Cell Death Detection Kit (Roche) following the manufacturer’s instructions. After counterstaining with DAPI, the oocytes were mounted on glass slides and examined with an LSM 900 laser scanning confocal microscope.

### Antibodies

The primary antibodies used in this study were anti-γH2AX (Abcam, ab111174, 1:500), anti-MDC1 (Abcam, ab241048, 1:100), anti-RAD51 (Abcam, ab63801, 1:100), anti-acetylated α-tubulin (Sigma–Aldrich, T7451, 1:500 or Abcam, ab179484, 1:500), anti-CIP2A (Santa Cruz, sc-80662, 1:500), anti-CENP-A (Cell Signaling, 2048S, 1:100), anti-CEP192 (Young in Frontier, AR07-PA0001, 1:200), anti-centromere (ACA; Antibodies Incorporated, 15-234, 1:100), anti-p-TOPBP1 (ABCEPTA, AP3774a-EV, 1:250), anti-BRCA1 (Abcam, ab17680, 1:100) and anti-pT210-PLK1 (Abcam, ab39068, 1:250). The secondary antibodies were Alexa Fluor 488-conjugated anti-mouse (Jackson ImmunoResearch, 115-545-146, 1:500), Alexa Fluor 488-conjugated anti-rabbit (Jackson ImmunoResearch, 111-545-003, 1:500), Alexa Fluor 594-conjugated anti-rabbit (Jackson ImmunoResearch, 111-585-045, 1:500) and rhodamine (TRITC)-conjugated anti-human (Jackson ImmunoResearch, 109-025-088, 1:100).

### Quantification of fluorescence intensity

All images were acquired at pixel dimensions of 1024 × 1024 and are shown as the maximum intensity of the Z-projections using an LSM 900 laser scanning confocal microscope. To measure immunofluorescence intensity, images were captured with identical laser power. The mean intensity of the fluorescence signals was measured, normalized to the mean DAPI or ACA signal intensity, and subsequently presented as arbitrary units. All data were analyzed using ZEN 3.4 Blue (Zeiss) and ImageJ software (National Institutes of Health) under the same processing parameters.

### Statistical analysis

Statistical analyses were performed using GraphPad Prism 9.0 (GraphPad Software). Data represent at least three independent experiments with each group containing at least 15 oocytes, unless specified otherwise. Differences between two groups were analyzed using Student’s *t*-test, and comparisons between more than two groups were analyzed using one-way analysis of variance and Tukey’s *post hoc* test. Correlation tests were analyzed using the Pearson correlation coefficient, with *r* < 1 representing a negative correlation. *P* < 0.05 was considered statistically significant.

## Results

### HR prevents chromosome fragmentation during meiotic maturation

Initially, we investigated the unique capacity of oocytes to repair DSBs during meiosis. To this end, we treated GV oocytes with ETP for 15 min and assessed γH2AX levels. After ETP treatment, γH2AX levels significantly increased, indicating the induction of DNA DSBs. Following ETP washout, we cultured these oocytes for 15 h to allow them to progress to the MII stage. Our results showed that γH2AX levels were abolished after 15 h of maturation (MII stage). However, substantial levels of γH2AX persisted at 4 h and peaked at 8 h, corresponding to the GVBD and MI stages, respectively (Supplementary Figure S1A and B). This aligned with the observation that MI oocytes typically exhibit high levels of γH2AX regardless of DNA damage (32). Thus, to further clarify the dynamics of DSB repair during meiotic maturation, we stained chromosomes with MDC1, a reliable DSB marker, as well as γH2AX (16). Our results showed a contrasting pattern to that observed for γH2AX, with MDC1 levels gradually decreasing during maturation (Supplementary Figure S1A and C), corroborating previous reports that oocytes could repair DSBs during meiotic maturation (15,16).

**Figure 1.**
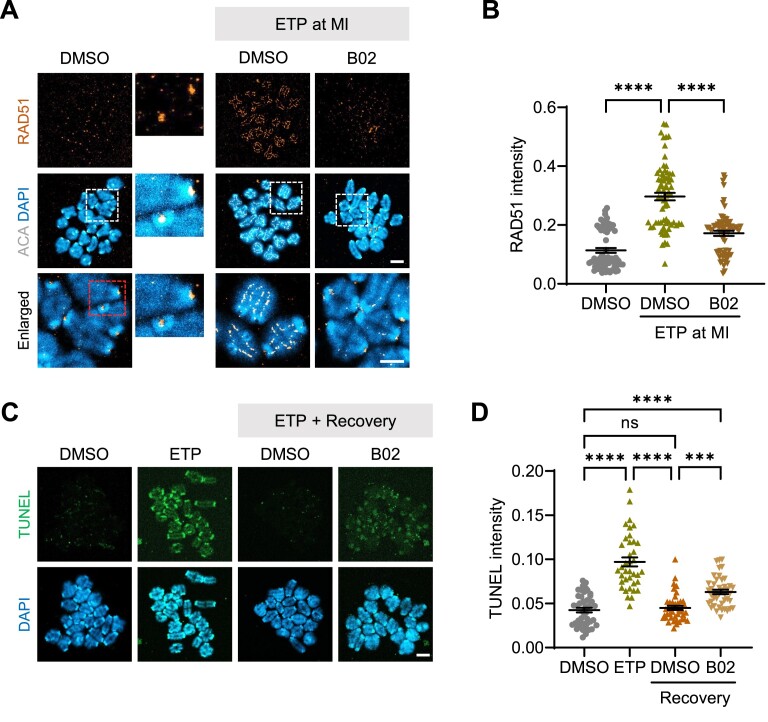
RAD51 inhibition using small molecule B02 impairs HR and DSB repair in oocytes. Oocytes at the MI stage were treated with ETP for 15 min with or without B02. Control oocytes were treated with DMSO. (**A**) Representative images of MI chromosomes stained with RAD51 and ACA antibodies. Scale bar, 10 μm. In the bottom panel of the DMSO control (enlarged), centromeres are highlighted with a dashed box and further magnified in the right panel. (**B**) Quantification of RAD51 intensity. (**C**) MI oocytes were treated with ETP for 15 min, washed out and cultured for 1 h to allow recovery from DNA damage. (**D**) Quantification of TUNEL intensity. Data are presented as the mean ± standard error of the mean (SEM) from three independent experiments. ****P < 0.0001, ***P < 0.0006; ns, not significant.

It is well established that the RAD51 is a key player in the HR pathway (2,3). To investigate whether HR is involved in DSB repair during meiotic maturation, we employed the small molecule B02, which inhibits RAD51 binding to single-stranded DNA and impairs HR (25,33), and examined RAD51 localization in response to DNA damage. We found that RAD51 signals appeared at centromeres but became enriched along the entire chromosomes after ETP treatment. Notably, this ETP-induced RAD51 enrichment on chromosomes was abolished by B02 treatment (Figure 1A and B). These results suggest that RAD51 is involved in DSB repair in oocytes, and that B02 specifically disrupts RAD51 recruitment on chromosomes, thereby inhibiting the HR pathway. To further validate the effectiveness of B02, we performed a TUNEL assay to assess the extent of DNA damage levels. We found that ETP treatment induced substantial levels of DNA damage, predominantly at centromeres, which significantly decreased after a 1-h recovery period. However, B02 treatment significantly decreased recovery from DNA damage, suggesting that HR actively participates in DSB repair in oocytes (Figure 1C and D).

**Figure 2.**
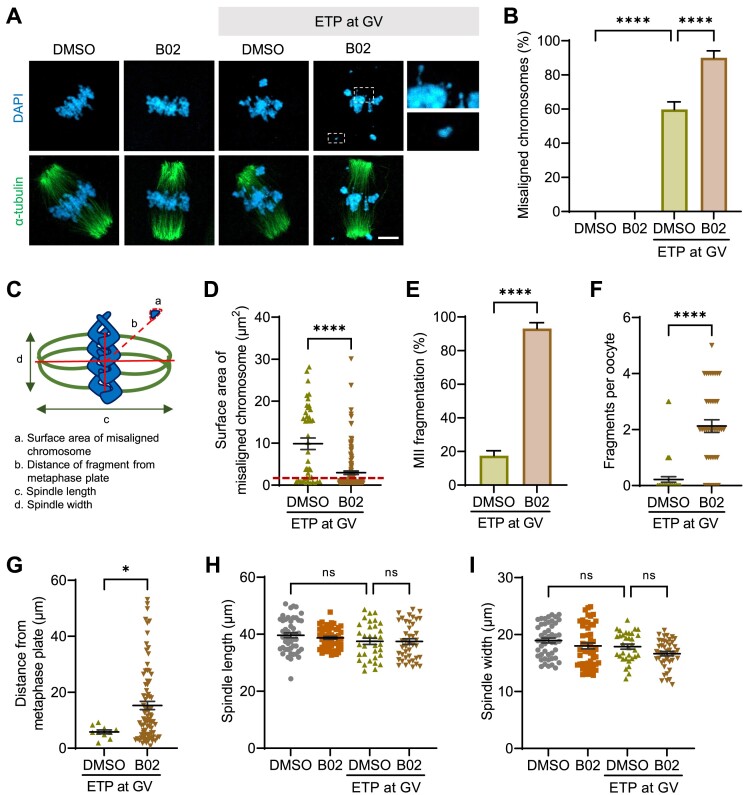
Inhibition of HR during meiotic maturation causes chromosome fragmentation following DSB induction. GV oocytes were treated with ETP for 15 min, washed out and matured to the MII stage in the presence of B02 for up to 15 h. Control oocytes were treated with DMSO instead of ETP and matured with or without B02. (**A**) Representative images of MII oocytes showing chromosome and spindle organization. Chromosome fragments in B02-treated oocytes are boxed and enlarged. Scale bar, 10 μm. (**B**) Quantification of the number of oocytes with misaligned chromosomes. (**C**) Schematic diagram of the parameters measured in the graphs (D–I). (**D**–**G**) Quantification of the surface area of misaligned chromosomes, MII fragmentation, number of fragments per oocyte and the distance of fragments from the center of the metaphase plate. (**H, I**) Quantification of spindle length and width. Data are presented as mean ± SEM of three independent experiments. ****P < 0.0001, *P < 0.03; ns, not significant.

To investigate the role of HR in DSB repair during meiotic maturation, we introduced DSBs to oocytes at the GV stage with ETP. Control oocytes were treated with DMSO instead of ETP. After ETP washout, we cultured these oocytes for 15 h to allow them to reach the MII stage, either in the presence or in the absence of B02, and examined chromosome and spindle organization. We confirmed that DMSO treatment had no effect on chromosome dynamics during meiotic maturation (Supplementary Figure S2). While control oocytes displayed well-aligned chromosomes regardless of B02 treatment during maturation, the ETP treatment led to an increase in chromosome misalignment, and this phenomenon was notably exacerbated after B02 treatment (Figure 2A and B). Intriguingly, in B02-treated oocytes, some misaligned chromosomes appeared fragmented and were observed outside of the spindle apparatus. To further clarify and distinguish these fragmented chromosomes from typical lagging chromosomes, we quantified their surface area and defined those measuring 1 μm^2^ or less as chromosome fragments (Figure 2C). According to this criterion, we noted a significantly higher proportion of B02-treated oocytes displaying chromosome fragmentation, along with an increased number of fragments per oocyte (Figure 2D–F). Moreover, these fragments exhibited greater dispersion from the metaphase plate in B02-treated oocytes (Figure 2G). Notably, spindle analysis revealed consistent spindle length and width across all experimental groups (Figure 2H and I), indicating that the observed phenotype was not attributable to spindle defects. Collectively, these findings suggest that RAD51 activity is required to repair DSBs and prevent chromosome fragmentation during meiotic maturation in oocytes.

### Chromosome fragmentation after HR inhibition is associated with impaired association of CIP2A on chromosomes

**Figure 3.**
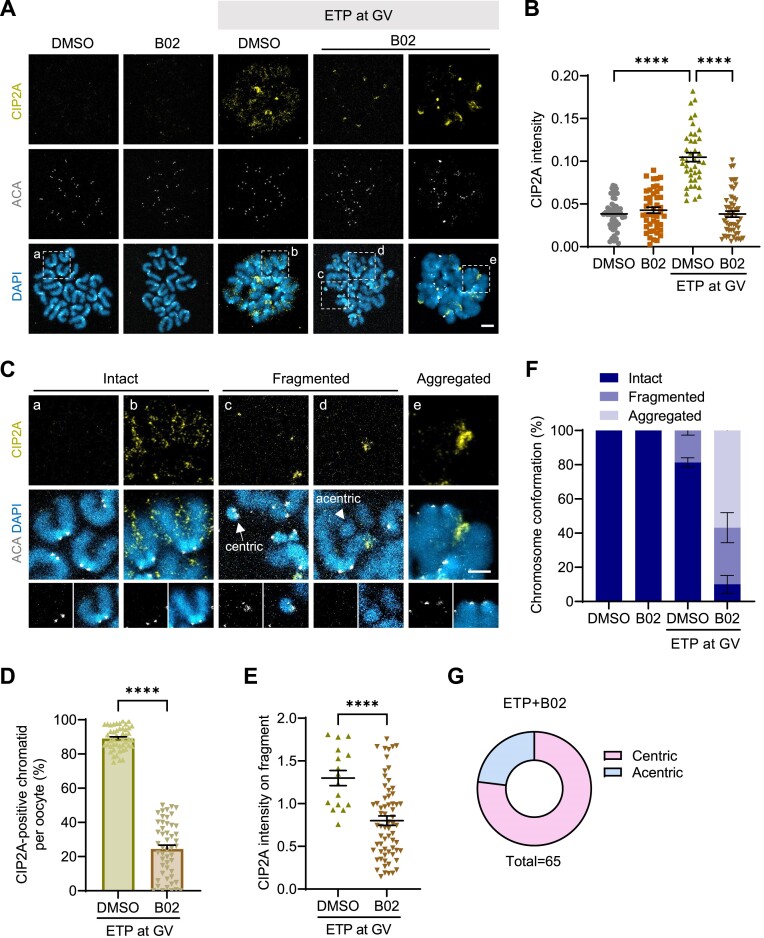
HR inhibition impairs the chromosomal association of CIP2A and disrupts chromosome conformation. GV oocytes were treated with ETP for 15 min, washed out and matured in the presence of B02 for up to 15 h. (**A**) Representative images of MII chromosomes stained with CIP2A and ACA antibodies. Scale bar, 10 μm. (**B**) Quantification of overall CIP2A intensity. (**C**) Enlarged views of the boxed regions (a–e) in panel (A). Chromosomes are categorized into three groups: intact, fragmented and aggregated. Centric and acentric fragments are marked with arrows and arrowheads, respectively. (**D**, **E**) Quantification of CIP2A-positive chromatid and CIP2A intensity on each fragment in ETP-treated groups. (**F**) Quantification of the types of chromosome conformations observed in each treatment group. (**G**) Distribution of centric and acentric fragments in B02-treated oocytes with DNA damage. Data are presented as mean ± SEM of three independent experiments. ****P< 0.0001.

We previously reported that the association of the CIP2A–MDC1–TOPBP1 complex with chromosomes is essential for DSB repair in mouse oocytes (16). Moreover, recent studies have revealed that the CIP2A–MDC1–TOPBP1 complex plays a critical role in clustering and tethering fragmented chromosomes in mitotic cells (20,21). Therefore, we sought to investigate whether the chromosome fragmentation observed in MII oocytes after HR inhibition is linked to the association of CIP2A with chromosomes. To test this possibility, we exposed GV oocytes to ETP, allowed them to mature for 15 h after ETP washout and examined the CIP2A levels on chromosomes at the MII stage. Our chromosome spread analyses showed increased CIP2A levels on chromosomes in oocytes exposed to ETP but significantly decreased levels by B02 treatment during meiotic maturation (Figure 3A and B). Most chromosome fragments observed in B02-treated oocytes were deficient in CIP2A signals, and when it was detected, signal intensity was significantly reduced compared to ETP-treated oocytes (Figure 3C–E). In all B02-treated oocytes, chromosome conformation could be largely categorized into either fragmented or aggregated. Fragmented chromosomes showed a partially maintained sister chromatid structure with visible fragments, whereas aggregated chromosomes displayed a complete breakdown of chromosome structure (Figure 3F). Interestingly, 78% of fragments derived from B02-treated oocytes were positive for ACA signals, indicating centric fragments (Figure 3G).

Given the dynamic changes in chromosome structure, including chromosome condensation and congression, we could not exclude the possibility that HR inhibition may disturb chromosome dynamics during meiotic maturation, indirectly affecting the chromosomal association of CIP2A. To address this concern, we introduced DSBs to MI oocytes and subsequently matured them to the MII stage after inhibiting HR. We found that ETP treatment significantly increased CIP2A levels on chromosomes. However, this increase in CIP2A levels was abrogated by B02 treatment, which was consistent with our results in GV oocytes (Supplementary Figure S3A and B). Taken together, our results suggest that chromosome fragmentations observed in HR-inhibited oocytes following exposure to ETP may be associated with impairment of CIP2A on chromosomes.

### HR regulates chromosomal recruitment of CIP2A

**Figure 4.**
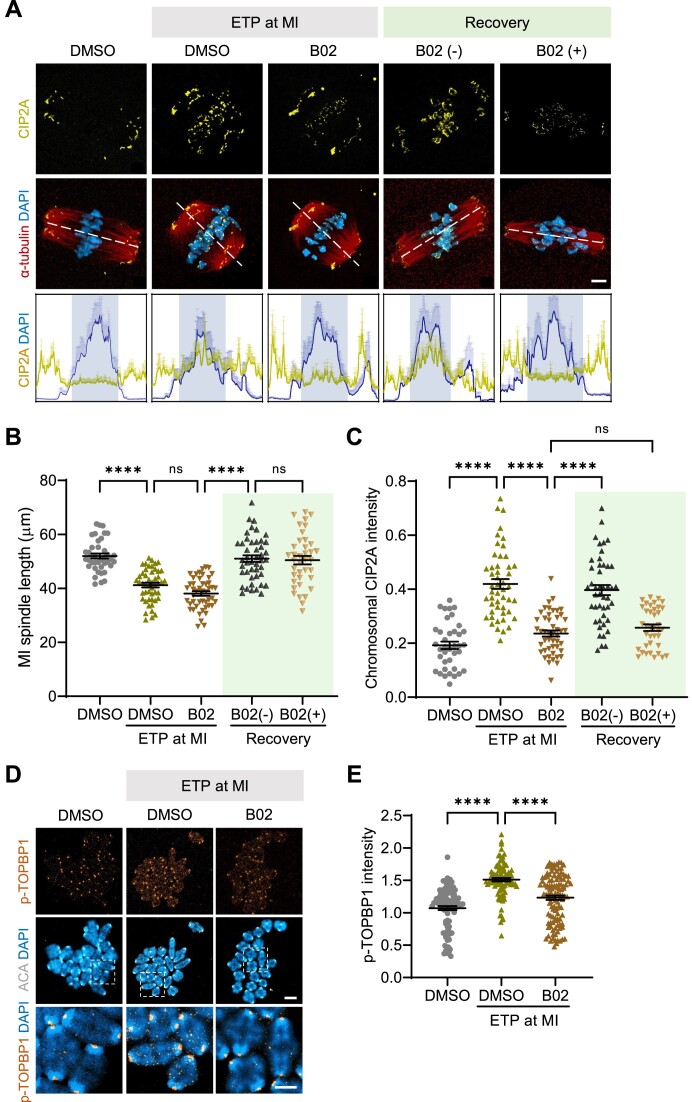
CIP2A recruitment onto damaged chromosomes is dependent on the HR pathway. Oocytes at the MI stage were treated with ETP in the presence of either DMSO or B02 for 15 min. After ETP washout, oocytes were allowed to recover for 1 h in the presence (+) or absence (−) of B02. (**A**) Representative images of MI oocytes showing CIP2A chromosomal recruitment. Profiling graphs are shown below to illustrate the pole-to-chromosome distribution of CIP2A. Scale bar, 10 μm. (**B**, **C**) Quantification of MI spindle length and chromosomal CIP2A intensity. (**D**) Representative images of MI chromosomes stained with p-TOPBP1 and ACA antibodies. Scale bar, 10 μm. (**E**) Quantification of p-TOPBP1 intensity. Data are presented as mean ± SEM of three independent experiments. ****P< 0.0001; ns, not significant.

In addition to chromosomal recruitment of CIP2A, spindle length is markedly reduced after DSB induction and restored during recovery from damage in MI oocytes (16). Given the similarity in the effects of ETP treatment on chromosome fragmentation and chromosomal association of CIP2A between GV and MI oocytes, we used MI oocytes to further investigate the connection between HR and CIP2A. We observed shrinkage and restoration of spindle lengths after ETP treatment and washout, respectively, consistent with our previous finding (16) (Figure 4A and B). Moreover, ETP treatment induced chromosomal recruitment of CIP2A. However, B02 treatment reduced the ETP-induced recruitment of CIP2A to the chromosomes (Figure 4A and C). Importantly, CIP2A reappeared on chromosomes after recovery following ETP and B02 washout. In contrast, the chromosomal recruitment of CIP2A was not rescued in the presence of B02 during recovery, suggesting that the chromosomal recruitment of CIP2A is dependent on the HR pathway (Figure 4A and C). As TOPBP1 is phosphorylated in response to DNA damage and collaborates with CIP2A as part of a tethering complex (16,34), we also examined p-TOPBP1 levels at the chromosomes and found a comparable decrease in p-TOPBP1 levels after B02 treatment (Figure 4D and E), further supporting the notion that the CIP2A–TOPBP1 tethering complex is reliant on the HR pathway.

Given that CIP2A recruitment to the chromosomes is dependent on the integrity of centromeres and microtubule-organizing centers (MTOCs) (16,35), it is possible that HR inhibition may affect the integrity of centromeres and MTOCs, thereby indirectly impairing CIP2A recruitment. To investigate this possibility, we examined CENP-A and CEP192 as markers for centromeres and MTOCs, respectively. However, we observed no discernible change in CENP-A or CEP192 levels after B02 treatment (Supplementary Figure S4A–E), excluding the possibility that HR inhibition affects the integrity of centromere and MTOCs.

**Figure 5.**
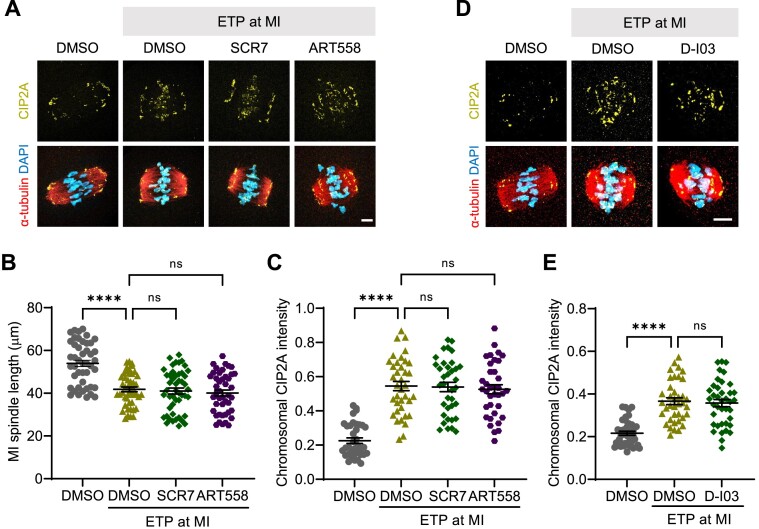
Chromosomal recruitment of CIP2A is independent of NHEJ, MMEJ and RAD52-dependent repair pathways. (**A**) Representative images of MI oocytes treated with ETP for 15 min in the presence of SCR7 (NHEJ inhibitor) or ART558 (MMEJ inhibitor). Oocytes were subjected to immunostaining with CIP2A antibody, showing chromosomal recruitment of CIP2A. Scale bar, 10 μm. (**B**, **C**) Quantification of MI spindle length and chromosomal CIP2A intensity. Data are presented as mean ± SEM of three independent experiments. ****P< 0.0001; ns, not significant. (**D**) Representative images of MI oocytes treated with ETP for 15 min in the presence of D-I03 (RAD52 inhibitor). Oocytes were subjected to immunostaining with CIP2A antibody, showing chromosomal recruitment of CIP2A. Scale bar, 10 μm. (**E**) Quantification of chromosomal CIP2A intensity. Data are presented as mean ± SEM of three independent experiments. ****P< 0.0001; ns, not significant.

In addition to HR, DSBs can be repaired by NHEJ and MMEJ (2,8). To investigate whether the chromosomal recruitment of CIP2A is also regulated by these pathways, we inhibited NHEJ by targeting DNA ligase IV using SCR7 (26). For MMEJ, we inhibited the key enzyme POLQ using ART588 (28). While ETP treatment induced spindle shrinkage, neither SCR7 nor ART588 prevented the ETP-induced CIP2A recruitment to the chromosomes (Figure 5A–C). Similar results were obtained when NHEJ was inhibited by targeting DNA-PK using NU7441 (27) (Supplementary Figure S5). This result suggests that NHEJ and MMEJ may not play a role in DSB-induced CIP2A recruitment to the chromosomes.

Although canonical DSB repair pathways are typically inactivated during mitosis (36), emerging evidence suggests that RAD52-dependent break-induced DNA replication can occur during mitosis and promote the repair of some DNA damage (37,38). To assess the possible role of RAD52 in DSB-induced CIP2A recruitment, we used the selective inhibitor D-I03, which specifically inhibits RAD52-dependent single-strand annealing and D-loop formation without affecting RAD51 foci formation (29). We found that D-I03 treatment did not affect ETP-induced CIP2A recruitment (Figure 5D and E), suggesting that RAD52-dependent repair pathways are unlikely to regulate CIP2A chromosomal recruitment. Therefore, our results collectively demonstrate that HR is required to recruit CIP2A on chromosomes, ensuring the preservation of genomic integrity during meiotic maturation in oocytes.

### CIP2A depletion causes chromosome fragmentation after DSB induction

**Figure 6.**
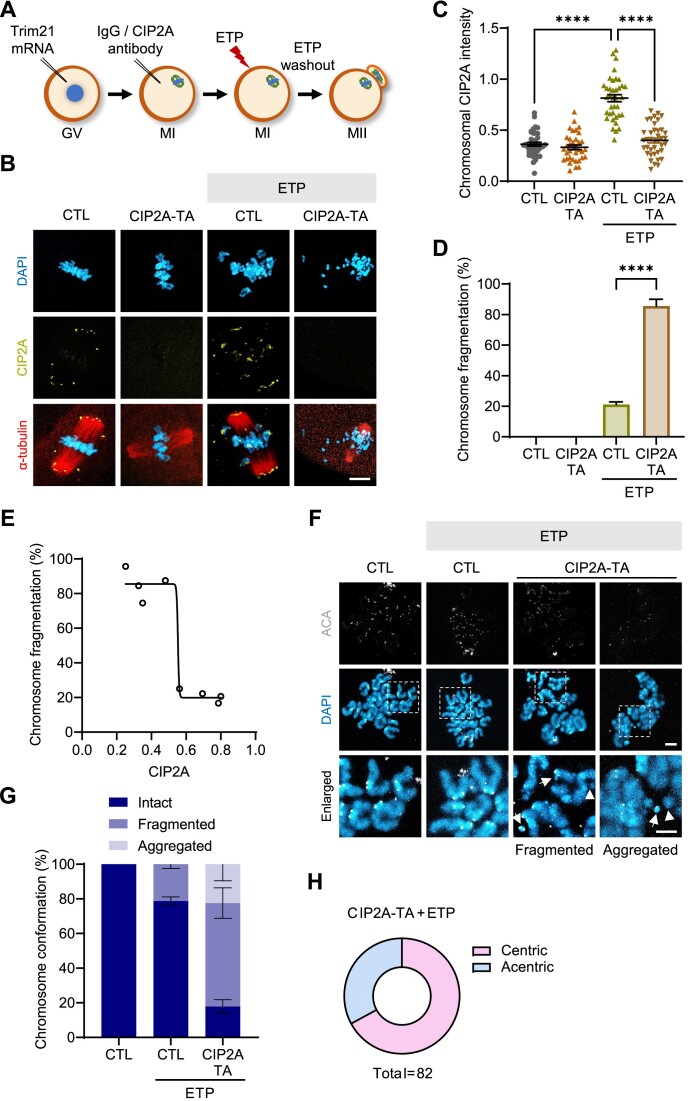
CIP2A depletion leads to chromosome fragmentation after DSB induction. (**A**) Scheme of Trim-away experiments to acutely deplete CIP2A in oocytes at the MI stage. GV oocytes were microinjected with Trim21-mCherry mRNA and cultured for 8 h to reach the MI stage. After confirming mCherry expression, MI oocytes were injected with either IgG control antibody (CTL) or CIP2A antibody (CIP2A-TA). After 1 h incubation to allow CIP2A depletion, oocytes were treated with ETP for 15 min, washed out and matured to the MII stage. (**B**) Representative images of MII oocytes showing chromosome fragmentations and CIP2A intensity. Scale bar, 10 μm. (**C**, **D**) Quantification of chromosomal CIP2A intensity and chromosome fragmentation. Data are presented as mean ± SEM of three independent experiments. ****P< 0.0001. (**E**) Correlation of CIP2A intensity against chromosome fragmentation after ETP treatment. *r* = −0.9091, *P*< 0.05. (**F**) Representative images of MII chromosomes illustrating chromosome fragmentation and aggregation. Centric and acentric fragments are marked with arrows and arrowheads, respectively. Scale bar, 10 μm. (**G**) Quantification of the types of chromosome conformations observed in each treatment group. (**H**) Distribution of centric and acentric fragments in CIP2A-depleted oocytes with DNA damage. Data are presented as mean ± SEM of three independent experiments.

To further investigate the protective role of CIP2A against chromosome fragmentation, we attempted to deplete CIP2A. Given that CIP2A depletion at the GV stage disrupts MTOC assembly and subsequent bipolar spindle formation leading to MI arrest (35), we opted for acute depletion of CIP2A via the Trim-away method at the MI stage when the bipolar spindles and kMT attachments are established (Figure 6A). We confirmed that our Trim-away strategy effectively depleted CIP2A without compromising spindle and chromosome organization (Figure 6B and C). Intact MTOC after CIP2A depletion was also confirmed by measuring CEP192 levels (Supplementary Figure S4F and G). After treatment with ETP, we allowed CIP2A-depleted oocytes to undergo meiotic maturation upon ETP washout. Our data showed a substantial increase in chromosome fragmentation in CIP2A-depleted oocytes compared to control oocytes injected with IgG (Figure 6B–D). Moreover, correlation analysis between chromosome fragmentations and CIP2A levels revealed a clear inverse relationship: as CIP2A levels decrease, fragmentation increases (Figure 6E). Notably, chromosome conformation analysis revealed that CIP2A depletion led to an increase in chromosome fragmentation and aggregation, with a notable abundance of centric fragments (Figure 6F–H). Interestingly, co-treatment with B02 further increased the proportion of aggregated chromosomes in CIP2A-depleted oocytes (Supplementary Figure S6), similar to the effects observed with B02 treatment alone (Figure 3F). Collectively, these findings suggest that CIP2A serves a tethering role to prevent chromosome fragmentation during meiotic maturation in oocytes.

### BRCA1 regulates chromosomal recruitment of CIP2A in response to DNA damage

**Figure 7.**
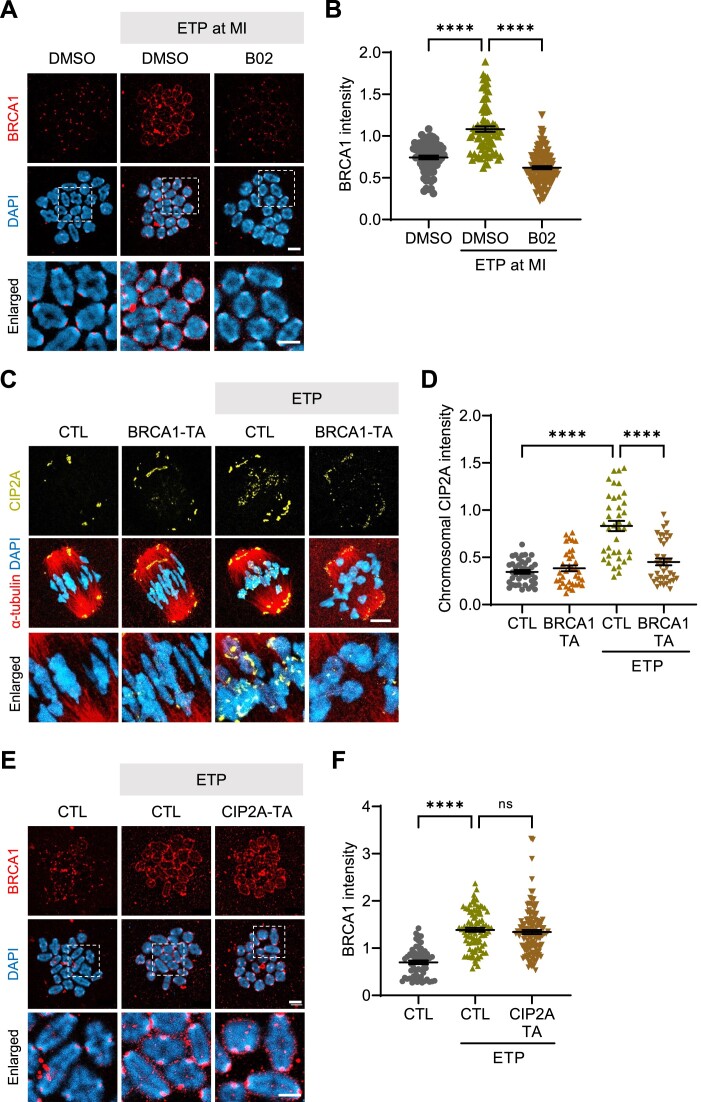
BRCA1 depletion impairs chromosomal recruitment of CIP2A induced by DNA damage. (**A**) Oocytes at the MI stage were treated with ETP in the presence of either DMSO or B02 for 15 min. Representative images of MI chromosomes stained with BRCA1 antibody. Scale bar, 10 μm. (**B**) Quantification of BRCA1 intensity. (**C**) Representative images of CIP2A chromosomal recruitment in MI oocytes after BRCA1 Trim-away (BRCA1-TA) and ETP treatment. BRCA1 Trim-away was conducted as described for CIP2A Trim-away. Scale bar, 10 μm. (**D**) Quantification of chromosomal CIP2A intensity. (**E**) Representative images of MI chromosomes stained with BRCA1 antibody after CIP2A Trim-away (CIP2A-TA). Scale bar, 10 μm. (**F**) Quantification of BRCA1 intensity. Data are presented as mean ± SEM of three independent experiments; ****P< 0.0001; ns, not significant.

BRCA1 is one of the key downstream factors that coordinate the HR pathway and is recruited to homologous chromosomes during meiotic maturation in oocytes after DNA damage (16). Thus, we investigated whether BRCA1 acts as an upstream HR component that regulates CIP2A recruitment in oocytes during the DDR. We found that BRCA1 levels increased after ETP treatment, and this increase was abrogated by B02 treatment, in a pattern consistent with that of CIP2A (Figure 7A and B). To further investigate the relationship between BRCA1 and CIP2A, we acutely depleted BRCA1 via Trim-away at the MI stage. After confirming successful depletion of BRCA1 (Supplementary Figure S7A and B), we examined CIP2A recruitment in response to DNA damage and found that ETP-induced CIP2A recruitment was significantly decreased after BRCA1 depletion (Figure 7C and D). To further clarify the relation between BRCA1 and CIP2A, we allowed the maturation of BRCA1-depleted oocytes to the MII stages after ETP washout and examined chromosome conformation and CIP2A levels. Similar to the effect of B02 treatment, BRCA1-depleted oocytes showed high levels of chromosome centric fragmentation and aggregation after ETP treatment (Supplementary Figure S7C–F). Moreover, the increase in CIP2A levels on chromosomes after treatment with ETP was abrogated by BRCA1 depletion (Supplementary Figure S7C and G). Also, most chromosome fragments were deficient in CIP2A signals or exhibited reduced intensity compared to control oocytes injected with IgG (Supplementary Figure S7C, H and I). Notably, unlike reduced CIP2A levels after BRCA1 depletion, CIP2A depletion did not yield any noticeable change in BRCA1 levels on chromosomes (Figure 7E and F), suggesting that CIP2A tethering operates downstream of BRCA1 on the DNA repair pathway.

### PLK1 inhibition restores impaired chromosomal recruitment of CIP2A mediated by inhibiting HR

**Figure 8.**
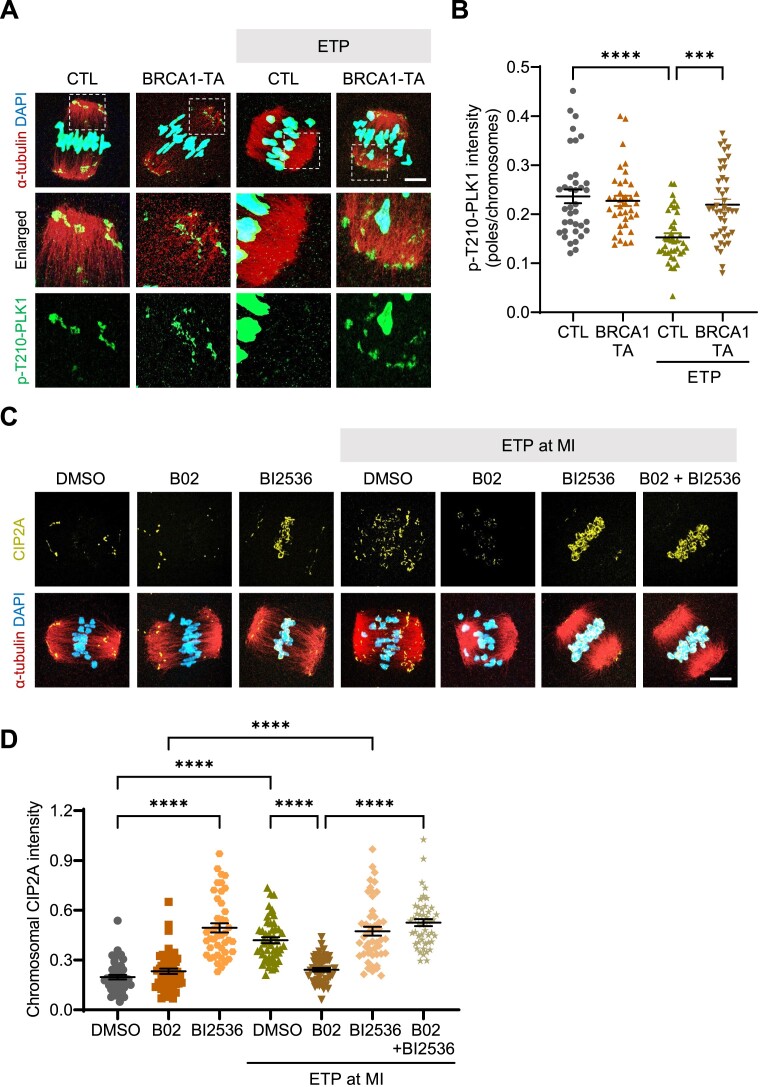
Impaired chromosomal recruitment of CIP2A induced by HR inhibition is restored by PLK1 inhibition. (**A**) Representative images of p-T210-PLK1 at spindle poles of MI oocytes after BRCA1 Trim-away (BRCA-TA). Scale bar, 10 μm. (**B**) Quantification of p-T210-PLK1 intensity at spindle poles. (**C**) Representative images of oocytes showing CIP2A chromosomal recruitment after treatment with ETP, B02 and BI2536. Scale bar, 10 μm. (**D**) Quantification of chromosomal CIP2A intensity. Data are presented as mean ± SEM of three independent experiments. ****P< 0.0001, ***P< 0.0006.

We have previously shown that DNA damage could induce PLK1 inactivation at the spindle poles, which promotes chromosomal recruitment of CIP2A in oocytes (16). This prompted us to investigate whether CIP2A recruitment induced by DNA damage is indirectly mediated by BRCA1 through PLK1 inactivation at the spindle poles. To investigate this, we examined phosphorylated PLK1 at T210 (p-T210-PLK1) at the spindle poles in BRCA1-depleted oocytes. Consistent with our previous finding, p-T210-PLK1 signals significantly decreased after ETP treatment. However, this ETP-induced decrease in p-T210-PLK1 signals at the spindle poles was abrogated in BRCA1-depleted oocytes (Figure 8A and B), suggesting that BRCA1 acts as an upstream regulator of PLK1 activity at the spindle poles in response to DNA damage. Similar results were observed when oocytes were treated with B02 (Supplementary Figure S8). To further clarify the relationship between BRCA1 and PLK1, we treated oocytes with the PLK1 inhibitor BI2536 in the presence of B02 and examined CIP2A recruitment after ETP treatment. Although B02 treatment compromised ETP-induced CIP2A recruitment, PLK1 inhibition rescued the chromosomal recruitment of CIP2A (Figure 8C and D). Taken together, our data illuminate the intricate interplay among BRCA1, PLK1 and CIP2A in orchestrating DSB repair to prevent chromosome fragmentation during meiotic maturation in oocytes.

## Discussion

This study discloses the critical role of the BRCA1–PLK1–CIP2A axis in safeguarding genomic integrity during meiotic maturation in oocytes. Our findings demonstrate that HR inhibition impedes the chromosomal association of CIP2A, leading to chromosome fragmentation. Furthermore, we provide evidence that BRCA1 and PLK1 act as upstream factors in the HR pathway that governs CIP2A recruitment to chromosomes upon DNA damage. These observations highlight the distinct strategies used by oocytes in repairing DSBs during meiosis.

Unlike mitotic cells, oocytes exhibit remarkable plasticity in DSB repair during meiotic maturation (12–17). Our data reinforce this notion by demonstrating that oocytes effectively repair DSBs induced by ETP during meiosis. We also found that HR inhibition during meiotic maturation partially disrupted this repair process, resulting in chromosome fragmentation, consistent with previous findings that oocytes selectively utilize both HR and NHEJ to repair DSBs during meiotic maturation (15). Our findings are in line with previous studies that emphasize the importance of the CIP2A–MDC1–TOPBP1 complex in tethering fragmented chromosomes in mitotic cells (20–23). We extend these observations to meiosis by demonstrating that CIP2A depletion phenocopies the effects of HR inhibition on chromosome conformation following DSB induction. This suggests that CIP2A plays a crucial role in repairing DSBs and preventing chromosome fragmentation during meiotic maturation in oocytes. Consistent with our findings, recent studies have shown that CIP2A knockdown causes dispersed chromosomes in human oocytes, and CIP2A loss-of-function mutation is associated with female infertility with early embryonic arrest and fragmentation (39).

We further elucidate the mechanism underlying HR-dependent recruitment of CIP2A on damaged chromosomes. Our data demonstrate that HR inhibition abrogates the ETP-induced recruitment of CIP2A to chromosomes. This suggests that HR signaling coordinates the mobilization of CIP2A to DSB sites for efficient repair. Interestingly, BRCA1 depletion phenocopied the effects of HR inhibition on CIP2A recruitment, whereas CIP2A depletion did not reduce BRCA1 recruitment to damaged chromosomes. Moreover, we found that chromosome aggregation was more severe in oocytes treated with B02 or depleted of BRCA1 than in those depleted of CIP2A. These results suggest not only that BRCA1 acts as an upstream regulator of CIP2A recruitment in the HR pathway during oocyte meiosis, but also that BRCA1 regulates chromosome integrity through additional pathways parallel with the CIP2A pathway.

In addition, we found that PLK1 is inactivated at the spindle poles in response to DNA damage, which is disrupted either by HR inhibition or by BRCA1 depletion. Given that PLK1-mediated phosphorylation is required for CIP2A localization at spindle poles in oocytes (35), BRCA1 may indirectly regulate CIP2A recruitment by modulating PLK1 activity in response to DNA damage. This notion is supported by the well-established relationship among BRCA1, PLK1 and CIP2A. It has been shown that CIP2A and PLK1 interact directly to facilitate spindle assembly and modulate cell cycle progression in cancer cells (40). Moreover, BRCA1 has been shown to downregulate PLK1 activity, whereas PLK1 is aberrantly activated when BRCA1 function is compromised (41,42). Taken together, our findings were the basis for a model wherein DSBs trigger BRCA1 recruitment to chromosomes during oocyte meiotic maturation. This activates the HR pathway and leads to inactivation of PLK1 at spindle poles, which facilitates recruitment of CIP2A to damaged chromosomes. Thus, CIP2A probably contributes to the HR-mediated DSB repair machinery during oocyte meiosis to safeguard genome integrity. Conversely, when either HR is inhibited or BRCA1 is depleted, PLK1 remains active at the spindle poles after DNA damage. Thus, CIP2A fails to recruit damaged chromosomes, causing chromosome fragmentation.

Recent studies identified CIP2A as a potential synthetic-lethal target in BRCA-mutated cancers, indicating its role in HR-deficient cells (22,23,43). However, our findings in oocytes suggest a different scenario, possibly due to inherent differences between mitosis and meiosis. Whereas mitotic cells suppress BRCA1 recruitment to damaged chromosomes and are refractory to DSB repair during mitosis (44–46), oocytes are capable of recruiting BRCA1 onto damaged chromosomes and undertaking DSB repair during meiosis (31). This inherent need for DSB repair during meiosis positions CIP2A as a potential downstream component within the HR machinery in oocytes. Notably, HR-deficient tumors overexpress and are highly dependent on POLQ (47). That TOPBP1 and POLQ are co-expressed with CIP2A suggests that POLQ supports the essential role of CIP2A in HR-deficient cells (43). In contrast, oocytes utilize HR and NHEJ to undertake DSB repair during meiosis (48). This discrepancy may explain why CIP2A depends on HR in oocytes, while it may cooperate with POLQ-dependent MMEJ in mitotic cells. However, we could not exclude the possibility that POLQ-dependent MMEJ might influence chromosomal recruitment of CIP2A under specific conditions or in the absence of a functional HR pathway in oocytes. The precise molecular mechanisms underlying the functional shift of CIP2A in oocytes remain to be determined. Future studies could explore potential differences in protein–protein interactions between oocytes and mitotic cells that govern the role of CIP2A in DSB repair.

In conclusion, this study uncovers a novel pathway by which BRCA1-dependent HR orchestrates CIP2A recruitment to chromosomes by regulating PLK1 activity, ensuring proper DSB repair and preventing chromosome fragmentation during meiotic maturation in oocytes. These findings not only provide insights into the unique mechanisms used by oocytes for meiotic repair, but also offer potential therapeutic targets for promoting healthy oocyte development and fertility in individuals with BRCA1 mutations.

## Supplementary Material

gkae1207_Supplemental_File

## Data Availability

All data needed to evaluate the conclusions in the paper are presented in the paper and/or the Supplementary Data. Additional data related to this paper can be requested from the authors.

## References

[1] Jackson S.P. , Bartek J. The DNA-damage response in human biology and disease. Nature. 2009; 461:1071–1078. 19847258 10.1038/nature08467 PMC2906700

[2] Shrivastav M. , De Haro L., Nickoloff J.A. Regulation of DNA double-strand break repair pathway choice. Cell Res. 2008; 18:134–147. 18157161 10.1038/cr.2007.111

[3] Ceccaldi R. , Rondinelli B., D’Andrea A.D. Repair pathway choices and consequences at the double-strand break. Trends Cell Biol. 2016; 26:52–64. 26437586 10.1016/j.tcb.2015.07.009 PMC4862604

[4] Rogakou E.P. , Pilch D.R., Orr A.H., Ivanova V.S., Bonner W.M. DNA double-stranded breaks induce histone H2AX phosphorylation on serine 139. J. Biol. Chem. 1998; 273:5858–5868. 9488723 10.1074/jbc.273.10.5858

[5] Leem J. , Lee C., Choi D.Y., Oh J.S. Distinct characteristics of the DNA damage response in mammalian oocytes. Exp. Mol. Med. 2024; 56:319–328. 38355825 10.1038/s12276-024-01178-2 PMC10907590

[6] Lieber M.R. The mechanism of double-strand DNA break repair by the nonhomologous DNA end-joining pathway. Annu. Rev. Biochem. 2010; 79:181–211. 20192759 10.1146/annurev.biochem.052308.093131 PMC3079308

[7] Li J. , Sun H., Huang Y., Wang Y., Liu Y., Chen X. Pathways and assays for DNA double-strand break repair by homologous recombination. Acta Biochim. Biophys. Sin. (Shanghai). 2019; 51:879–889. 31294447 10.1093/abbs/gmz076

[8] Wang H. , Xu X. Microhomology-mediated end joining: new players join the team. Cell Biosci. 2017; 7:6. 28101326 10.1186/s13578-017-0136-8 PMC5237343

[9] Mateos-Gomez P.A. , Gong F., Nair N., Miller K.M., Lazzerini-Denchi E., Sfeir A. Mammalian polymerase theta promotes alternative NHEJ and suppresses recombination. Nature. 2015; 518:254–257. 25642960 10.1038/nature14157 PMC4718306

[10] Holt J.E. , Lane S.I., Jones K.T. The control of meiotic maturation in mammalian oocytes. Curr. Top. Dev. Biol. 2013; 102:207–226. 23287034 10.1016/B978-0-12-416024-8.00007-6

[11] Carroll J. , Marangos P. The DNA damage response in mammalian oocytes. Front. Genet. 2013; 4:117. 23805152 10.3389/fgene.2013.00117 PMC3690358

[12] Collins J.K. , Lane S.I., Merriman J.A., Jones K.T. DNA damage induces a meiotic arrest in mouse oocytes mediated by the spindle assembly checkpoint. Nat. Commun. 2015; 6:8553. 26522232 10.1038/ncomms9553 PMC4659839

[13] Leem J. , Kim J.S., Oh J.S. WIP1 phosphatase suppresses the DNA damage response during G2/prophase arrest in mouse oocytes. Biol. Reprod. 2018; 99:798–805. 29733326 10.1093/biolre/ioy108

[14] Martin J.H. , Bromfield E.G., Aitken R.J., Lord T., Nixon B. Double strand break DNA repair occurs via non-homologous end-joining in mouse MII oocytes. Sci. Rep. 2018; 8:9685. 29946146 10.1038/s41598-018-27892-2 PMC6018751

[15] Lee C. , Leem J., Oh J.S. Selective utilization of non-homologous end-joining and homologous recombination for DNA repair during meiotic maturation in mouse oocytes. Cell Prolif. 2023; 56:e13384. 36564861 10.1111/cpr.13384 PMC10068936

[16] Leem J. , Kim J.S., Oh J.S. Oocytes can repair DNA damage during meiosis via a microtubule-dependent recruitment of CIP2A–MDC1–TOPBP1 complex from spindle pole to chromosomes. Nucleic Acids Res. 2023; 51:4899–4913. 36999590 10.1093/nar/gkad213 PMC10250218

[17] Leem J. , Bai G.Y., Kim J.S., Oh J.S. Melatonin protects mouse oocytes from DNA damage by enhancing nonhomologous end-joining repair. J. Pineal Res. 2019; 67:e12603. 31370106 10.1111/jpi.12603

[18] Zhang C.Z. , Spektor A., Cornils H., Francis J.M., Jackson E.K., Liu S., Meyerson M., Pellman D. Chromothripsis from DNA damage in micronuclei. Nature. 2015; 522:179–184. 26017310 10.1038/nature14493 PMC4742237

[19] Stephens P.J. , Greenman C.D., Fu B., Yang F., Bignell G.R., Mudie L.J., Pleasance E.D., Lau K.W., Beare D., Stebbings L.A. et al. Massive genomic rearrangement acquired in a single catastrophic event during cancer development. Cell. 2011; 144:27–40. 21215367 10.1016/j.cell.2010.11.055 PMC3065307

[20] Lin Y.F. , Hu Q., Mazzagatti A., Valle-Inclan J.E., Maurais E.G., Dahiya R., Guyer A., Sanders J.T., Engel J.L., Nguyen G. et al. Mitotic clustering of pulverized chromosomes from micronuclei. Nature. 2023; 618:1041–1048. 37165191 10.1038/s41586-023-05974-0 PMC10307639

[21] Trivedi P. , Steele C.D., Au F.K.C., Alexandrov L.B., Cleveland D.W. Mitotic tethering enables inheritance of shattered micronuclear chromosomes. Nature. 2023; 618:1049–1056. 37316668 10.1038/s41586-023-06216-z PMC10424572

[22] Adam S. , Rossi S.E., Moatti N., De Marco Zompit M., Xue Y., Ng T.F., Alvarez-Quilon A., Desjardins J., Bhaskaran V., Martino G. et al. The CIP2A–TOPBP1 axis safeguards chromosome stability and is a synthetic lethal target for BRCA-mutated cancer. Nat. Cancer. 2021; 2:1357–1371. 35121901 10.1038/s43018-021-00266-w

[23] De Marco Zompit M. , Esteban M.T., Mooser C., Adam S., Rossi S.E., Jeanrenaud A., Leimbacher P.A., Fink D., Shorrocks A.K., Blackford A.N. et al. The CIP2A–TOPBP1 complex safeguards chromosomal stability during mitosis. Nat. Commun. 2022; 13:4143. 35842428 10.1038/s41467-022-31865-5 PMC9288427

[24] Xiong B. , Li S., Ai J.S., Yin S., Ouyang Y.C., Sun S.C., Chen D.Y., Sun Q.Y. BRCA1 is required for meiotic spindle assembly and spindle assembly checkpoint activation in mouse oocytes. Biol. Reprod. 2008; 79:718–726. 18596218 10.1095/biolreprod.108.069641

[25] Huang F. , Motlekar N.A., Burgwin C.M., Napper A.D., Diamond S.L., Mazin A.V. Identification of specific inhibitors of human RAD51 recombinase using high-throughput screening. ACS Chem. Biol. 2011; 6:628–635. 21428443 10.1021/cb100428c PMC3117970

[26] Srivastava M. , Nambiar M., Sharma S., Karki S.S., Goldsmith G., Hegde M., Kumar S., Pandey M., Singh R.K., Ray P. et al. An inhibitor of nonhomologous end-joining abrogates double-strand break repair and impedes cancer progression. Cell. 2012; 151:1474–1487. 23260137 10.1016/j.cell.2012.11.054

[27] Leahy J.J. , Golding B.T., Griffin R.J., Hardcastle I.R., Richardson C., Rigoreau L., Smith G.C. Identification of a highly potent and selective DNA-dependent protein kinase (DNA-PK) inhibitor (NU7441) by screening of chromenone libraries. Bioorg. Med. Chem. Lett. 2004; 14:6083–6087. 15546735 10.1016/j.bmcl.2004.09.060

[28] Zatreanu D. , Robinson H.M.R., Alkhatib O., Boursier M., Finch H., Geo L., Grande D., Grinkevich V., Heald R.A., Langdon S. et al. Polθ inhibitors elicit BRCA-gene synthetic lethality and target PARP inhibitor resistance. Nat. Commun. 2021; 12:3636. 34140467 10.1038/s41467-021-23463-8 PMC8211653

[29] Huang F. , Goyal N., Sullivan K., Hanamshet K., Patel M., Mazina O.M., Wang C.X., An W.F., Spoonamore J., Metkar S. et al. Targeting BRCA1- and BRCA2-deficient cells with RAD52 small molecule inhibitors. Nucleic Acids Res. 2016; 44:4189–4199. 26873923 10.1093/nar/gkw087 PMC4872086

[30] Clift D. , So C., McEwan W.A., James L.C., Schuh M. Acute and rapid degradation of endogenous proteins by Trim-away. Nat. Protoc. 2018; 13:2149–2175. 30250286 10.1038/s41596-018-0028-3

[31] Kim M. , Kim J., Yang S., Lee D.W., Park S.G., Leem J.H., Kim H.C. The relationship between fatigue and sickness absence from work. Ann. Occup. Environ. Med. 2023; 35:e32. 37701492 10.35371/aoem.2023.35.e32 PMC10493375

[32] Mayer A. , Baran V., Sakakibara Y., Brzakova A., Ferencova I., Motlik J., Kitajima T.S., Schultz R.M., Solc P. DNA damage response during mouse oocyte maturation. Cell Cycle. 2016; 15:546–558. 26745237 10.1080/15384101.2015.1128592 PMC5056612

[33] Huang F. , Mazina O.M., Zentner I.J., Cocklin S., Mazin A.V. Inhibition of homologous recombination in human cells by targeting RAD51 recombinase. J. Med. Chem. 2012; 55:3011–3020. 22380680 10.1021/jm201173g

[34] Yamane K. , Wu X., Chen J. A DNA damage-regulated BRCT-containing protein, TopBP1, is required for cell survival. Mol. Cell. Biol. 2002; 22:555–566. 11756551 10.1128/MCB.22.2.555-566.2002 PMC139754

[35] Wang H. , Choe M.H., Lee I.W., Namgoong S., Kim J.S., Kim N.H., Oh J.S. CIP2A acts as a scaffold for CEP192-mediated microtubule organizing center assembly by recruiting Plk1 and aurora A during meiotic maturation. Development. 2017; 144:3829–3839. 28935709 10.1242/dev.158584

[36] Bakhoum S.F. , Kabeche L., Compton D.A., Powell S.N., Bastians H. Mitotic DNA damage response: at the crossroads of structural and numerical cancer chromosome instabilities. Trends Cancer. 2017; 3:225–234. 28718433 10.1016/j.trecan.2017.02.001 PMC5518619

[37] Minocherhomji S. , Ying S., Bjerregaard V.A., Bursomanno S., Aleliunaite A., Wu W., Mankouri H.W., Shen H., Liu Y., Hickson I.D. Replication stress activates DNA repair synthesis in mitosis. Nature. 2015; 528:286–290. 26633632 10.1038/nature16139

[38] Bhowmick R. , Minocherhomji S., Hickson I.D. RAD52 facilitates mitotic DNA synthesis following replication stress. Mol. Cell. 2016; 64:1117–1126. 27984745 10.1016/j.molcel.2016.10.037

[39] Liu Z. , Xi Q., Hou M., Zou T., Liu H., Zhou X., Jin L., Zhu L., Zhang X. Loss of function variant in CIP2A associated with female infertility with early embryonic arrest and fragmentation. Biochim. Biophys. Acta Mol. Basis Dis. 2024; 1870:167228. 38734318 10.1016/j.bbadis.2024.167228

[40] Kim J.S. , Kim E.J., Oh J.S., Park I.C., Hwang S.G. CIP2A modulates cell-cycle progression in human cancer cells by regulating the stability and activity of Plk1. Cancer Res. 2013; 73:6667–6678. 23983103 10.1158/0008-5472.CAN-13-0888

[41] He Z. , Ghorayeb R., Tan S., Chen K., Lorentzian A.C., Bottyan J., Aalam S.M.M., Pujana M.A., Lange P.F., Kannan N. et al. Pathogenic BRCA1 variants disrupt PLK1-regulation of mitotic spindle orientation. Nat. Commun. 2022; 13:2200. 35459234 10.1038/s41467-022-29885-2 PMC9033786

[42] Zou J. , Rezvani K., Wang H., Lee K.S., Zhang D. BRCA1 downregulates the kinase activity of Polo-like kinase 1 in response to replication stress. Cell Cycle. 2013; 12:2255–2265. 24067368 10.4161/cc.25349 PMC3755076

[43] Laine A. , Nagelli S.G., Farrington C., Butt U., Cvrljevic A.N., Vainonen J.P., Feringa F.M., Gronroos T.J., Gautam P., Khan S. et al. CIP2A interacts with TopBP1 and drives basal-like breast cancer tumorigenesis. Cancer Res. 2021; 81:4319–4331. 34145035 10.1158/0008-5472.CAN-20-3651 PMC8373817

[44] Leimbacher P.A. , Jones S.E., Shorrocks A.K., de Marco Zompit M., Day M., Blaauwendraad J., Bundschuh D., Bonham S., Fischer R., Fink D. et al. MDC1 interacts with TOPBP1 to maintain chromosomal stability during mitosis. Mol. Cell. 2019; 74:571–583. 30898438 10.1016/j.molcel.2019.02.014 PMC6509287

[45] Giunta S. , Belotserkovskaya R., Jackson S.P. DNA damage signaling in response to double-strand breaks during mitosis. J. Cell Biol. 2010; 190:197–207. 20660628 10.1083/jcb.200911156 PMC2930281

[46] Nelson G. , Buhmann M., von Zglinicki T. DNA damage foci in mitosis are devoid of 53BP1. Cell Cycle. 2009; 8:3379–3383. 19806024 10.4161/cc.8.20.9857

[47] Ceccaldi R. , Liu J.C., Amunugama R., Hajdu I., Primack B., Petalcorin M.I., O’Connor K.W., Konstantinopoulos P.A., Elledge S.J., Boulton S.J. et al. Homologous-recombination-deficient tumours are dependent on Polθ-mediated repair. Nature. 2015; 518:258–262. 25642963 10.1038/nature14184 PMC4415602

[48] Kim H.D. , Kim H.C., Leem J.H. Toxic hepatitis after exposure to humidifier disinfectant: a case series report. Environ. Anal. Health Toxicol. 2023; 38:e2023002-0. 37100397 10.5620/eaht.2023002 PMC10195676

