# Supplementary material for: Novel BRCA1–PLK1–CIP2A axis orchestrates homologous recombination-mediated DNA repair to maintain chromosome integrity during oocyte meiosis
Source: Nucleic Acids Res. 2024 Dec 9;53(2):gkae1207. doi: 10.1093/nar/gkae1207 (PMC11754672; doi:10.1093/nar/gkae1207)
Supplement: gkae1207_Supplemental_File [file gkae1207_supplemental_file.pdf]

## Fig. S1

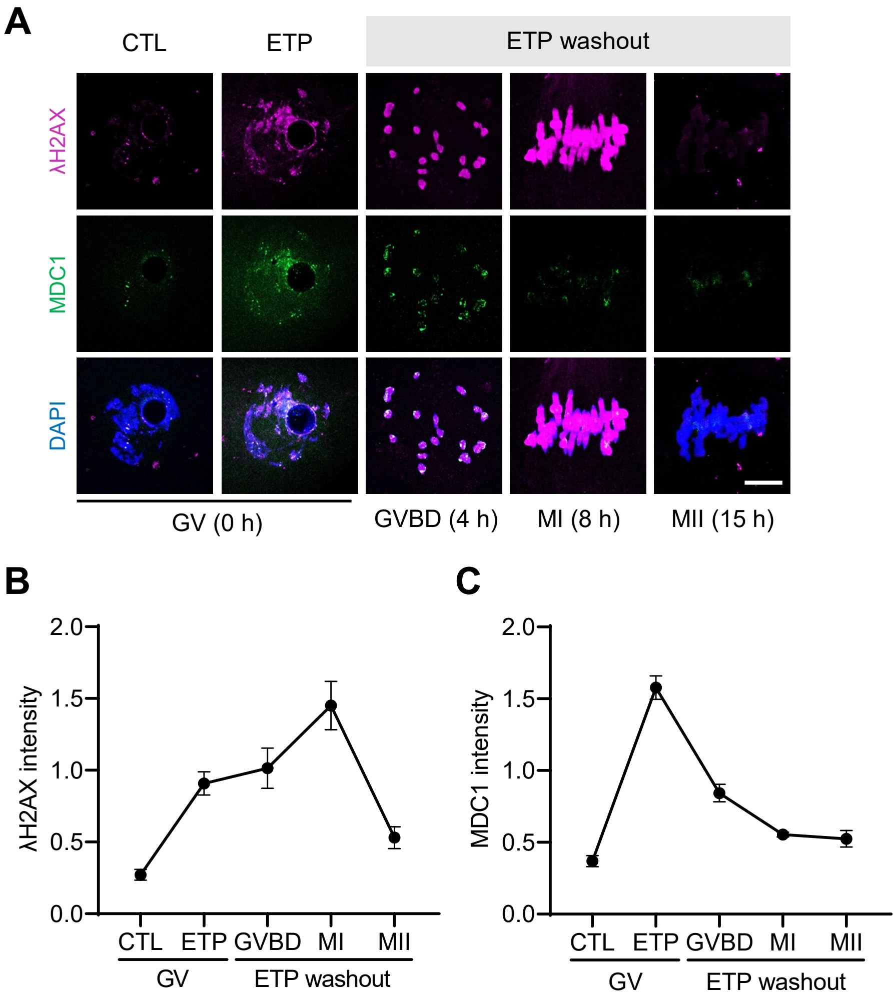


**Fig. S1. Oocytes are capable of repairing DNA damage during meiotic maturation**. Oocytes at the GV stage were treated with ETP for 15 min, washed, and allowed to mature for up to 15 h to reach the MII stage. Oocytes were collected at 0, 4, 8, and 15 h of culture in IBMX-free medium, corresponding to the GV, GVBD, MI, and MII stages, respectively, and subjected to immunostaining analysis. **(A)** Representative images of oocytes at GV, GVBD, MI, and MII stages, stained with γH2AX and MDC1 antibodies. Scale bar, 10 μm. **(B, C)** Quantification of γH2AX and MDC1 intensities. Data are presented as mean ± SEM of two independent experiments.

**Fig. S2**

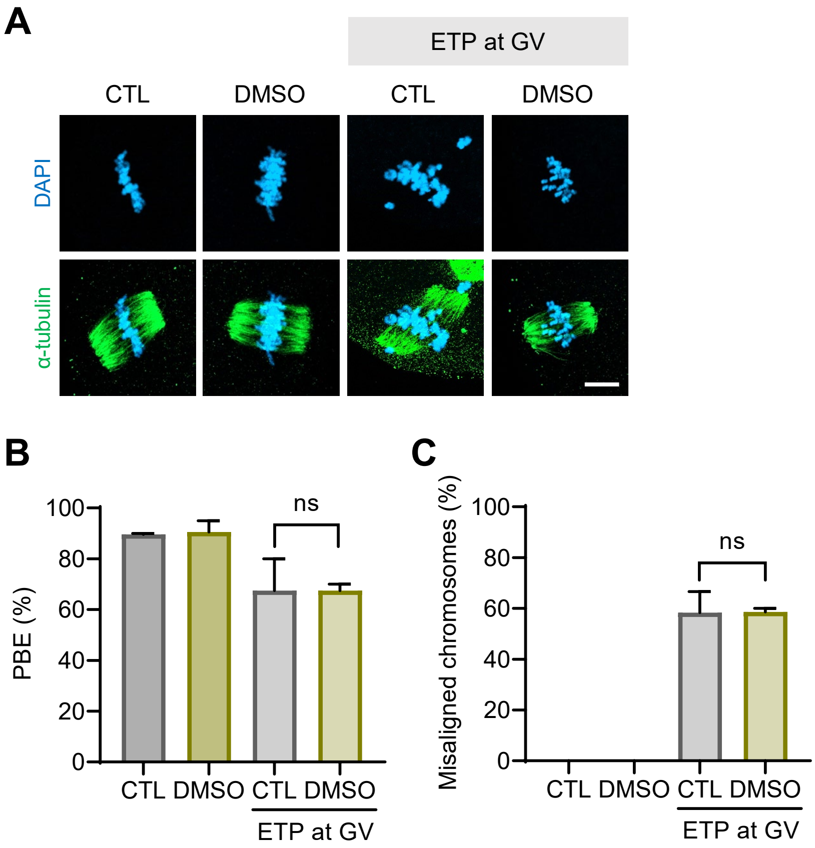


**Fig. S2. Negligible effect of DMSO on chromosome dynamics during meiotic maturation**. Oocytes at the GV stage were treated with ETP for 15 min, washed, and allowed to mature for up to 15 h to reach the MII stage. To assess the impact of DMSO, oocytes were either untreated (control; CTL) or treated with DMSO. **(A)** Representative images of MII oocytes showing chromosome and spindle organization. Scale bar, 10 μm. **(B)** Rate of the polar body extrusion (PBE). **(C)** Quantification of the number of oocytes with misaligned chromosomes. Data are presented as mean ± SEM of three independent experiments. ns, not significant.

## Fig. S3

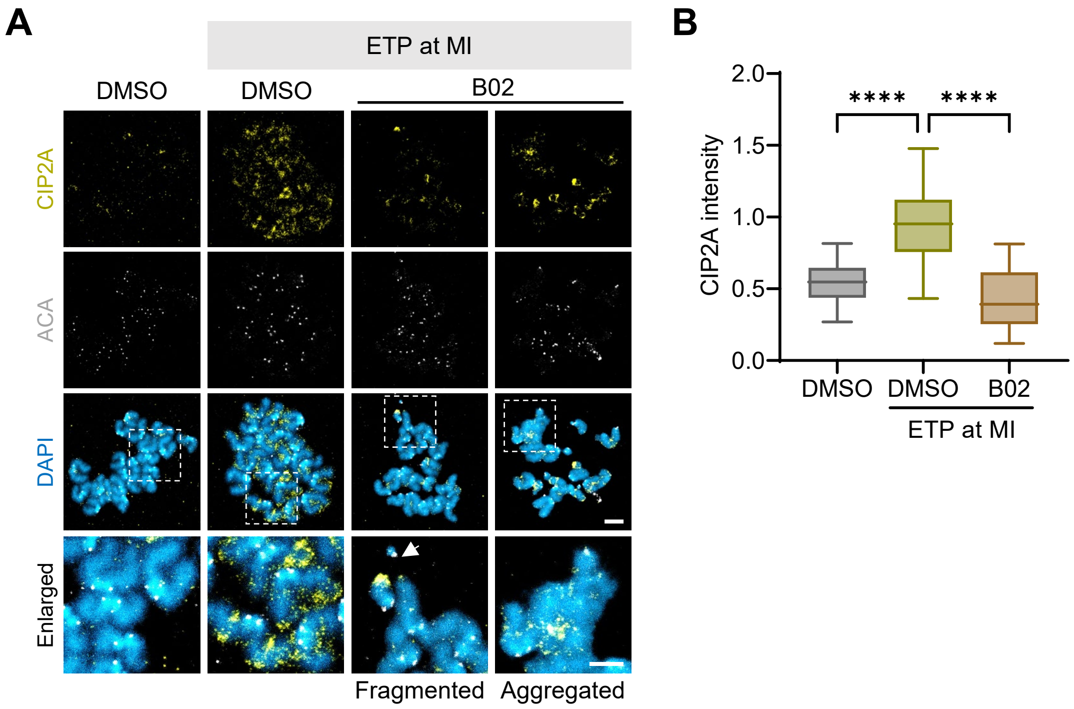


**Fig. S3. HR inhibition impairs chromosomal association of CIP2A when ETP is treated at the MI stage.** Oocytes were matured to the MI stage, treated with ETP for 15 min, followed by ETP washout, and matured in the presence of B02 for up to 15 h. **(A)** Representative images of MII chromosomes stained with CIP2A and ACA antibodies. Scale bar, 10 μm. Centric fragment is marked with arrow. **(B)** Quantification of CIP2A intensity. Data are presented as mean ± SEM of two independent experiments. ****$p$ < 0.0001.

## Fig. S4

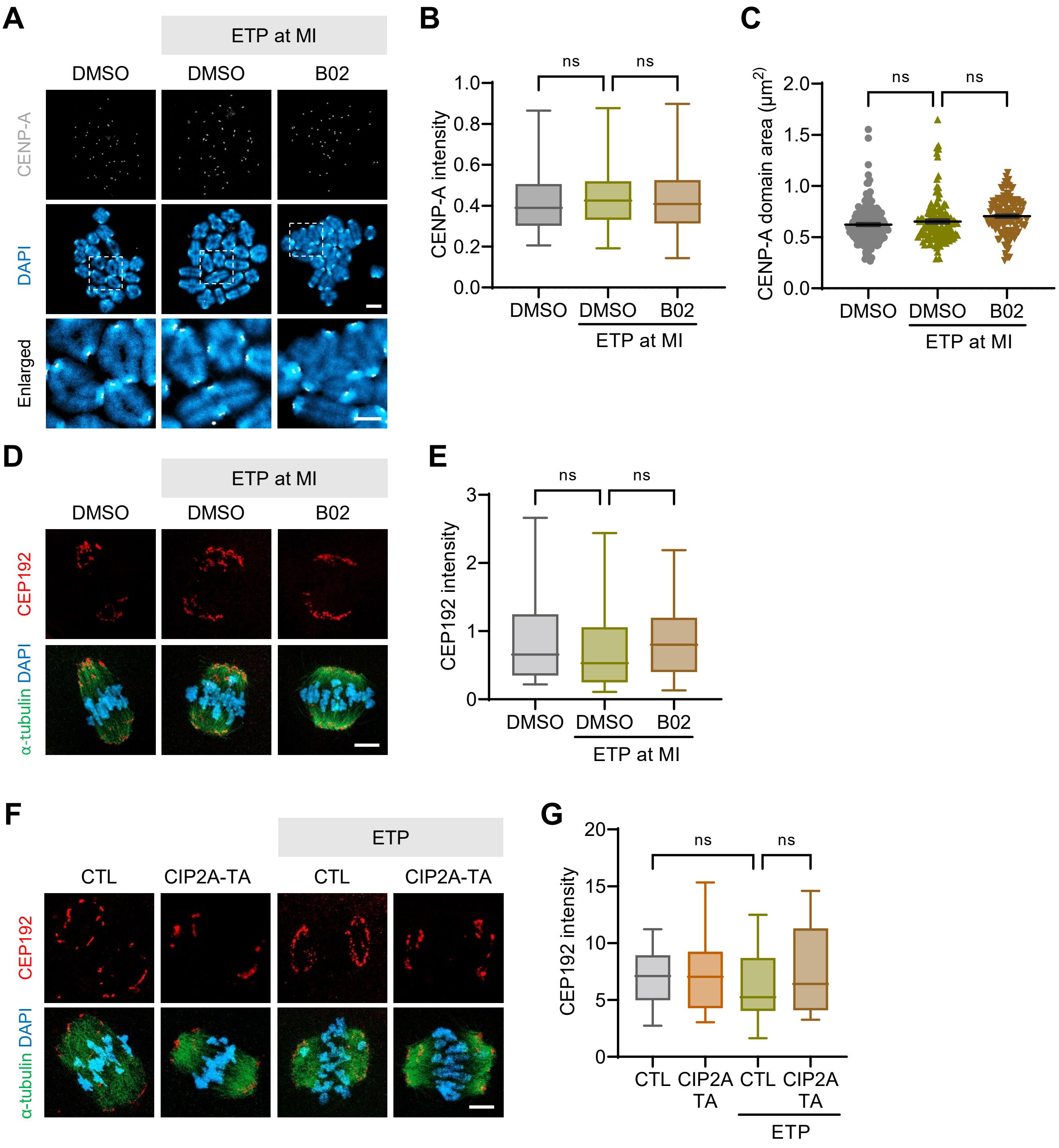


**Fig. S4. B02 treatment and CIP2A Trim-away do not affect centromeres or MTOC integrity. (A)** Representative images of MI chromosomes stained with CENP-A antibody. Scale bar, 10 μm. **(B, C)** Quantification of CENP-A intensity and domain area. **(D)** Representative images of MI oocytes showing CEP192 levels at the spindle poles. Scale bar, 10 μm. **(E, G)** Quantification of CEP192 intensity at spindle poles. ns, not significant. **(F)** Representative images of control (CTL) or CIP2A Trim-away (CIP2A-TA) oocytes showing CEP192 levels at the spindle pole. Scale bar, 10 μm.

## Fig. S5

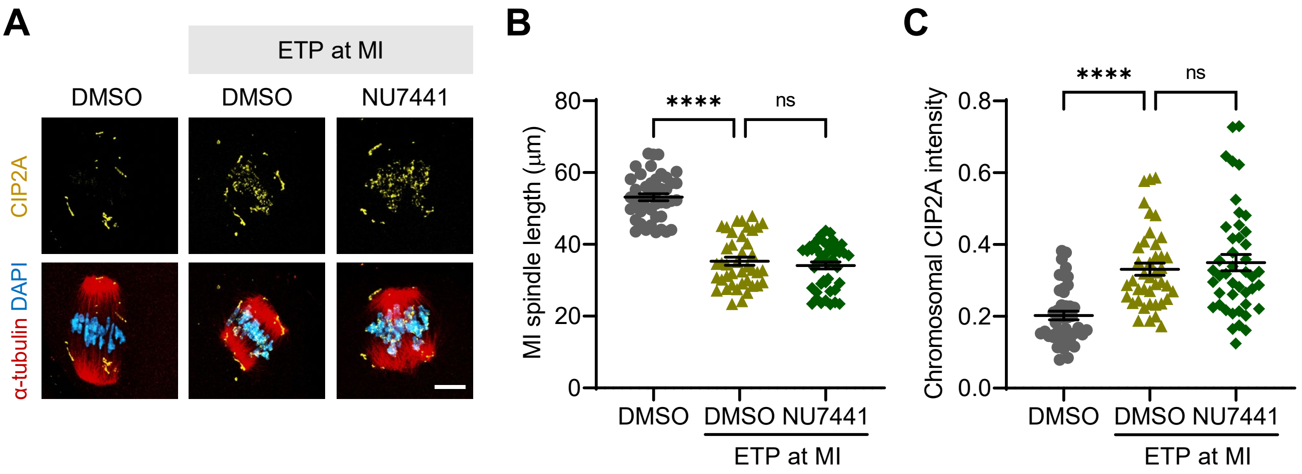


**Fig. S5. Inhibition of DNA-PK using NU1774 does not impair chromosomal recruitment of CIP2A after DNA damage**. **(A)** Representative images of MI oocytes treated with ETP for 15 min in the presence of NU1774. Oocytes were subjected to immunostaining with CIP2A antibody, showing chromosomal relocation of CIP2A. Scale bar, 10 μm. **(B, C)** Quantification of MI spindle length and chromosomal CIP2A intensity. Data are presented as the mean ± SEM from three independent experiments. ****$p < 0.0001$; ns, not significant.

## Fig. S6

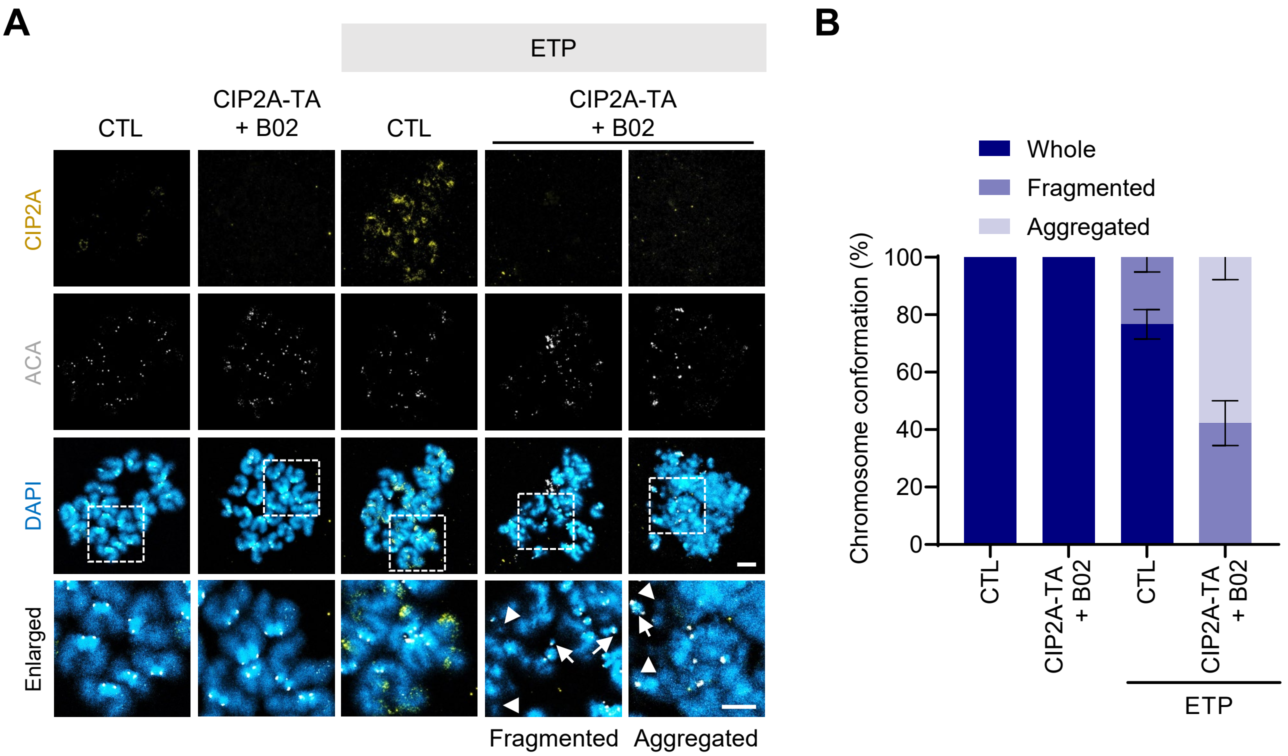


**Fig. S6. B02 treatment further increases chromosomal aggregation induced by CIP2A depletion.** GV oocytes were microinjected with Trim21-mCherry mRNA and cultured for 8 h to reach the MI stage. After confirming mCherry expression, MI oocytes were injected with either IgG control antibody (CTL) or CIP2A antibody (CIP2A-TA). After 1 h incubation to allow CIP2A depletion, oocytes were treated with ETP for 15 min, washed out, and matured to the MII stage with B02. **(A)** Representative images of MII chromosomes illustrating chromosome fragmentation and aggregation. Centric and acentric fragments are marked with arrows and arrowheads, respectively. Scale bar, 10 μm. **(B)** Quantification of the types of chromosome conformations observed in each treatment group. Data are presented as mean ± SEM of three independent experiments.

## Fig. S7

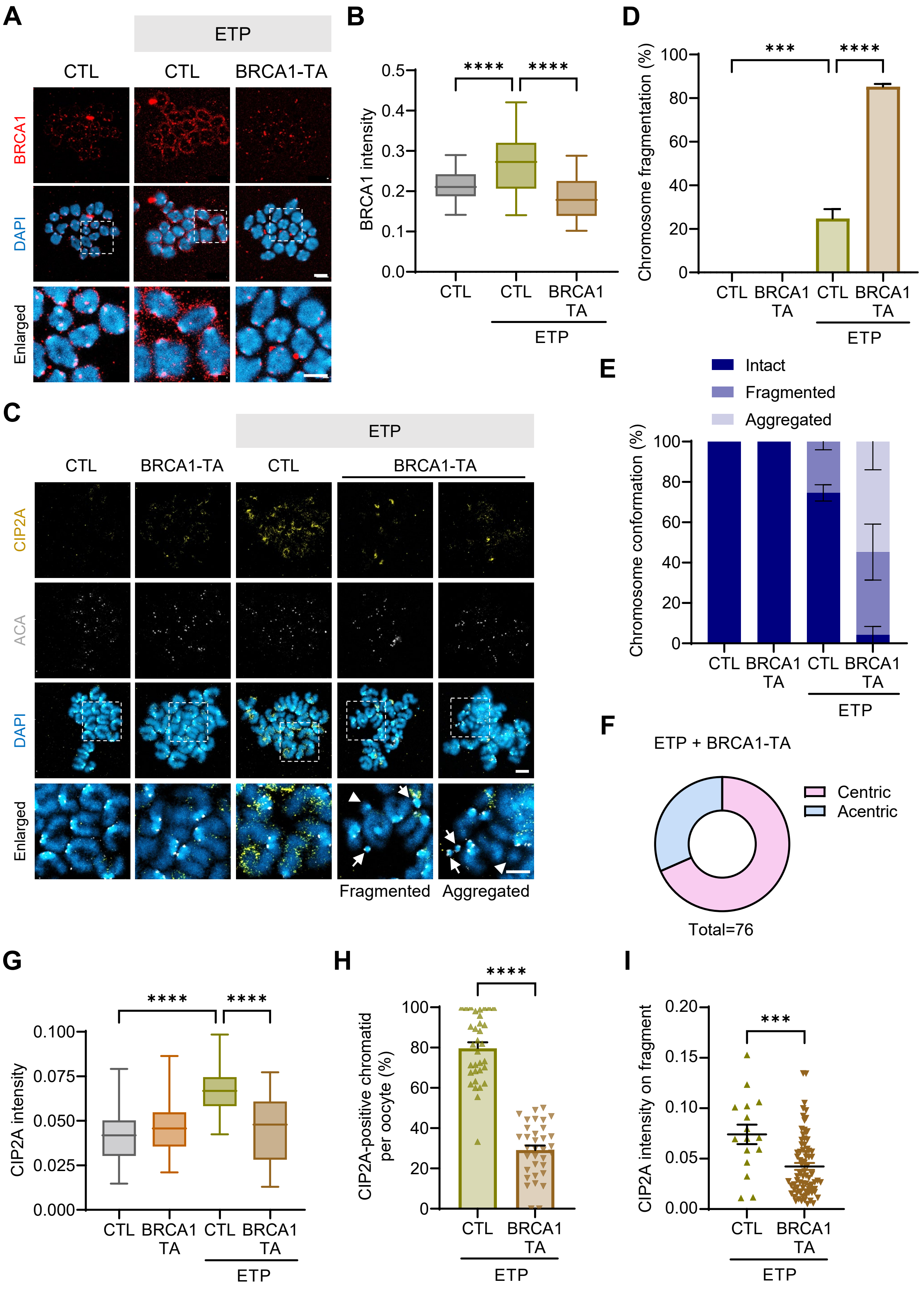

A
ETP
CTL
CTL
BRCA1-TA
BRCA1
DAPI
Enlarged
B
BRCA1 intensity
0.0
0.1
0.2
0.3
0.4
0.5
****
****
CTL
CTL
BRCA1 TA
ETP
D
Chromosome fragmentation (%)
0
20
40
60
80
100
***
****
CTL
BRCA1 TA
CTL
BRCA1 TA
ETP
C
ETP
CTL
BRCA1-TA
CTL
BRCA1-TA
CIP2A
ACA
DAPI
Enlarged
Fragmented
Aggregated
E
Intact
Fragmented
Aggregated
Chromosome conformation (%)
0
20
40
60
80
100
CTL
BRCA1 TA
CTL
BRCA1 TA
ETP
F
ETP + BRCA1-TA
Centric
Acentric
Total=76
G
CIP2A intensity
0.000
0.025
0.050
0.075
0.100
****
****
CTL
BRCA1 TA
CTL
BRCA1 TA
ETP
H
CIP2A-positive chromatid per oocyte (%)
0
20
40
60
80
100
****
CTL
BRCA1 TA
ETP
I
CIP2A intensity on fragment
0.00
0.05
0.10
0.15
0.20
***
CTL
BRCA1 TA
ETP

**Fig. S7. BRCA1 depletion mimics the effects of CIP2A depletion.** GV oocytes were microinjected with Trim21-mCherry mRNA and cultured for 8 h to reach the MI stage. After confirming mCherry expression, MI oocytes were injected with either IgG control antibody (CTL) or BRCA1 antibody (BRCA1-TA). After 1 h incubation to allow BRCA1 depletion, oocytes were treated with ETP for 15 min and either immediately subjected to chromosome spreading at the MI stage (A, B) or matured to the MII stage after ETP washout (C-I). **(A)** Representative images of MI chromosomes stained with BRCA1 antibody after ETP treatment. Scale bar, 10 μm. **(B)** Quantification of BRCA1 intensity. **(C)** Representative images of MII chromosomes stained with CIP2A and ACA antibodies. Centric and acentric fragments are marked with arrows and arrowheads, respectively. Scale bar, 10 μm. **(D)** Quantification of chromosome fragmentation. **(E)** Quantification of the types of chromosome conformations observed in each treatment group. **(F)** Distribution of centric and acentric fragments in BRCA1-depleted oocytes with DNA damage. **(G-I)** Quantification of CIP2A intensity, CIP2A-positive chromatid, and CIP2A intensity on each fragment in ETP-treated groups. Data are presented as mean ± SEM of three independent experiments. ****$p < 0.0001$, ***$p < 0.0006$.

**Fig. S8**

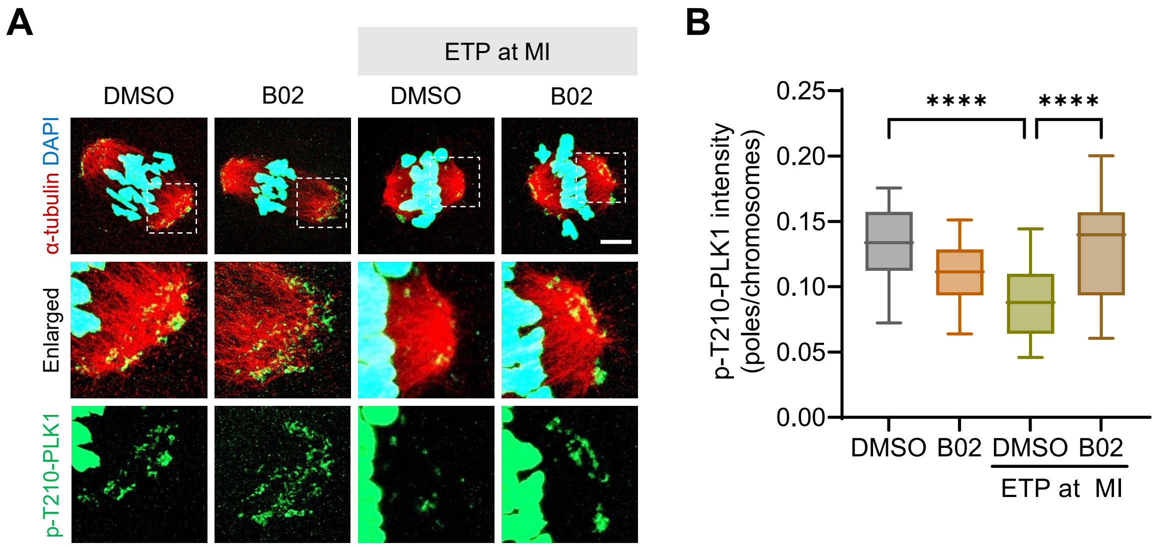


**Fig. S8. HR inhibition impairs DNA damage-induced PLK1 inactivation at the spindle poles. (A)** Representative images of MI oocytes stained with p-T210-PLK1 antibody after ETP and B02 treatment. **(B)** Quantification of p-T210-PLK1 intensity at spindle poles. Data are presented as mean ± SEM of three independent experiments. ****$p < 0.0001$.
